# Molecular characterization of carbapenem-resistant *Escherichia coli* and *Acinetobacter baumannii* in the Lao People’s Democratic Republic

**DOI:** 10.1093/jac/dkz234

**Published:** 2019-06-05

**Authors:** Tomas-Paul Cusack, Vilayouth Phimolsarnnousith, Khuanta Duangmala, Phonelavanh Phoumin, Jane Turton, Katie L Hopkins, Neil Woodford, Nandini Shetty, Nicole Stoesser, Hang T T Phan, David A B Dance

**Affiliations:** 1 Lao-Oxford-Mahosot Hospital-Wellcome Trust Research Unit (LOMWRU), Microbiology Laboratory, Mahosot Hospital, Vientiane, Lao PDR; 2 National Infection Service, Public Health England, London, UK; 3 Nuffield Department of Clinical Medicine, Oxford University, John Radcliffe Hospital, Headington, UK; 4 National Institute for Health Research (NIHR) Oxford Biomedical Research Centre, John Radcliffe Hospital, Headington, UK; 5 Centre for Tropical Medicine and Global Health, Nuffield Department of Medicine, University of Oxford, Oxford, UK; 6 Faculty of Infectious and Tropical Diseases, London School of Hygiene and Tropical Medicine, London, UK

Sir,

Global dissemination of carbapenemases among Gram-negative bacteria is a growing public health concern. Therapeutic options for these organisms are often limited, with alternative agents such as tigecycline and colistin having potentially less favourable efficacy and toxicity profiles.^1^^,^^2^ Furthermore, these agents are expensive and not readily available in resource-constrained settings. In the Lao People’s Democratic Republic (Laos), carbapenems are not yet on the national list of essential drugs, although in Vientiane the high prevalence of ESBL-producing Enterobacterales^3^^,^^4^ has driven more widespread use of carbapenems imported by individual pharmacies from neighbouring countries. However, while carbapenem-resistant Enterobacterales (CRE) and carbapenem-resistant *Acinetobacter baumannii* (CRAB) have been described in neighbouring Thailand and Vietnam,^5^ little is known about carbapenem resistance in Laos, where few laboratories perform antimicrobial susceptibility testing (AST) and surveillance networks are not well established.

The Microbiology Laboratory, Mahosot Hospital, Vientiane, Laos, receives clinical samples from several hospitals in Vientiane and other provinces, undertakes AST using CLSI methodology and participates in the UK National External Quality Assessment (UK NEQAS) scheme for AST. Since 2010, isolates of Enterobacterales and *Acinetobacter* spp. displaying resistance to three or more first-line agents have been routinely tested against meropenem using 10 μg discs (Oxoid, Basingstoke, UK). In 2017, 280/428 *Escherichia coli*, 67/208 *Klebsiella pneumoniae* and 35/111 *Acinetobacter* spp. isolates underwent meropenem susceptibility testing. The first carbapenem-resistant *E. coli* was identified in 2015. A second isolate (Patient 2) was sent to the Oxford Genomics Centre (University of Oxford, Oxford, UK), where WGS using the Illumina HiSeq 2500 platform identified it as *E. coli* ST410 carrying *bla*_NDM-5_, prompting the current review.

Laboratory records were retrospectively reviewed for meropenem- or imipenem-resistant Enterobacterales (from 1 January 2010 to 31 December 2017) and *Acinetobacter* spp. (from 1 January to 31 December 2017). All CRE and CRAB were retrieved from storage at −80°C and their identity was confirmed using API 20E for Enterobacterales and API 20NE for *Acinetobacter* spp. (bioMérieux, Basingstoke, UK). Phenotypic susceptibilities were confirmed by disc diffusion according to CLSI 2018 standards and breakpoints,^6^ and the modified carbapenem inactivation method (mCIM)^6^ was used to detect carbapenemase production. Clinical and demographic data were obtained from review of hospital charts. The isolates were sent for further characterization at PHE (Colindale, London, UK), where they were tested for *bla*_KPC_, *bla*_OXA-48-like_, *bla*_NDM_, *bla*_VIM_, *bla*_IMP_, *bla*_SIM_, *bla*_GIM_, *bla*_SPM_, *bla*_FRI_, *bla*_IMI_, *bla*_GES_ and *bla*_SME_ carbapenemase genes and *mcr*-1/-2 acquired colistin resistance genes using the AusDiagnostics MT CRE EU assay (AusDiagnostics, Chesham, UK).^7^ CRAB were also tested for *bla*_OXA-58-like_, *bla*_OXA-23-like_, *bla*_OXA-51-like_ and *bla*_OXA-40-like_ carbapenemase genes using a previously described OXA/class 1 integrase gene/*rpoB* multiplex PCR.^8^

Four CRE isolates, all *E. coli*, were identified from four patients from two hospitals in Vientiane (1 from 2015, 1 from 2016 and 2 from 2017) (Table 1). Two were isolated from urine, one from a wound swab, and one from a blood culture. All were resistant to all β-lactams tested as well as to ciprofloxacin, gentamicin, co-trimoxazole and tetracycline. Three isolates were susceptible to amikacin, and both urinary isolates were also susceptible to fosfomycin and nitrofurantoin, but the bloodstream isolate was only susceptible to doxycycline and nitrofurantoin. None of the patients had documented prior exposure to carbapenems. The mCIM test confirmed carbapenemase production and molecular analysis identified a New Delhi MBL (NDM) gene in all four isolates. NDM subtype was not determined.

**Table 1:** Clinical and microbiological details of four carbapenem-resistant *E. coli* isolated at Mahosot Microbiology Laboratory

Parameter	Patient 1	Patient 2	Patient 3	Patient 4
Age (years)	33	50	55	56
Sex	female	male	male	female
Specimen	abdominal wound swab	urine	blood culture	urine
Specimen date	August 2015	September 2016	July 2017	July 2017
Ward	gastrointestinal surgery	urology	general medicine	urology
Reason for admission/ clinical syndrome	wound infection and liver abscess post-cholecystectomy	right perinephric abscess post-renal tract surgery in Savannakhet province for calculi	biliary sepsis, underlying cholangiocarcinoma	pyelonephritis associated with ureteric calculus
Phenotypic AST results				
susceptible	AMK, CHL, FOF^a^	AMK, FOF, NIT	DOX, NIT^a^	AMK, FOF, NIT
intermediate	NIT^a^			
resistant	AMP, AMC, CAZ, CIP, CPD, CRO, DOX, GEN, IPM, MEM, SXT, TET	AMP, AMC, CAZ, CIP, CPD, CRO, DOX, GEN, IPM, MEM, SXT, TET	AMK, AMP, AMC, C, CAZ, CIP, CPD, CRO, FOF^a^, GEN, IPM, MEM, SXT, TET	AMP, AMC, CAZ, CIP, CPD, CRO, DOX, GEN, IPM, MEM, SXT, TET
Acquired carbapenemase genes detected	*bla*_NDM_	*bla*_NDM_ ^b^	*bla*_NDM_	*bla*_NDM_
Carbapenem exposure prior to specimen collection	no	no	no	no
Status at discharge	alive, well	alive, well	alive, re-admitted August 2017 with recurrent fever	moribund

AMK, amikacin; AMC, amoxicillin/clavulanate; AMP, ampicillin; CAZ, ceftazidime; CHL, chloramphenicol; CIP, ciprofloxacin; GEN, gentamicin; CPD, cefpodoxime; CRO, ceftriaxone; DOX, doxycycline; NIT, nitrofurantoin; FOF, fosfomycin; IPM, imipenem; MEM, meropenem; SXT, trimethoprim/sulfamethoxazole; TET, tetracycline.

a Zone diameter interpreted according to CLSI criteria for urinary isolates.

b Previously confirmed as bla_NDM-5_ by WGS.

Meropenem resistance was confirmed in 22 non-duplicate *Acinetobacter* spp. isolates in 2017, all of which contained *bla*_OXA-51-like_ genes intrinsic to *A. baumannii* (Table S1, available as Supplementary data at *JAC* Online). All isolates were also resistant to ceftriaxone, ceftazidime, imipenem, ciprofloxacin and tetracycline. Eighteen (81.8%) were susceptible to amikacin. Two isolates were not susceptible to any agents tested. All CRAB produced the OXA-23-like carbapenemase, with two additionally carrying an NDM carbapenemase gene. Most CRAB (19/22) were from endotracheal aspirates from the adult ICU at Mahosot Hospital, but, as isolates were not further characterized, we could not determine whether this reflected cross-infection in the ICU or the emergence of multiple independent strains. Although colistin susceptibility was not tested phenotypically, *mcr-1/-2* genes were not detected in either species.

To the best of our knowledge, this is the first report of carbapenemase-producing *E. coli* and *A. baumannii* in Laos. While our results are from a single laboratory and therefore may not be representative of the epidemiology of carbapenem resistance nationally, Mahosot Hospital is a tertiary referral centre from other provinces and the Microbiology Laboratory also receives specimens from hospitals in several provinces, comprising an informal surveillance network. Molecular findings are consistent with reports from Thailand and Vietnam, where carbapenem resistance in *A. baumannii* and Enterobacterales appears to be predominantly related to OXA-23-like carbapenemases and NDM carbapenemases, respectively.^5^

In summary, this study demonstrates the presence of OXA-23-like and NDM carbapenemases in Laos. Given the increasing use of carbapenems, lack of established infection control protocols, and limited access to alternative therapeutic agents in Laos, this is of grave concern. Efforts to prevent further dissemination of these organisms in Laos must be prioritized.

## Funding

The Lao-Oxford-Mahosot Hospital-Wellcome Trust Research Unit (LOMWRU) is core funded by Wellcome (grant number 106698/Z/14/Z). The funding body had no role in: the design of the study; collection, analysis and interpretation of data; and writing of the manuscript. Study-related work at the other sites was supported by internal funding.

## Transparency declarations

None to declare.

## Supplementary Material

dkz234_Supplementary_Data
