# Supplementary material for: Molecular characterization of carbapenem-resistant Escherichia coli and Acinetobacter baumannii in the Lao People’s Democratic Republic
Source: J Antimicrob Chemother. 2019 Jun 5;74(9):2810–21. doi: 10.1093/jac/dkz234 (PMC6736291; doi:10.1093/jac/dkz234)
Supplement: dkz234_Supplementary_Data [file dkz234_supplementary_data.docx]

**Supplementary data**

**Table S1.** Demographic and microbiological data for 22 non-duplicate carbapenem-resistant *Acinetobacter baumannii* isolated at Mahosot Microbiology Laboratory in 2017

| **Patient** | **Age** | **Sex** | **Specimen** | **Ward (Hospital)** | **Phenotypic AST** | | | **Carbapenem exposure prior to specimen collection** | **Acquired carbapenemase genes detected** |
| --- | --- | --- | --- | --- | --- | --- | --- | --- | --- |
|  |  |  |  |  | **S** | **I** | **R** |  |  |
| AB1 | 91 | F | ETA | ICUA (MH) | AMK, GEN | SAM, SXT | CAZ, CIP, CRO, DOX, IPM, MEM, TET | NO | *bla*_OXA-23-like_ |
| AB2 | 57 | M | ETA | ICUA (MH) | AMK, GEN | SXT | CAZ, CIP, CRO, DOX, IPM, MEM, SAM, TET | NO | *bla*_OXA-23-like_ |
| AB3 | 55 | M | ETA | ICUA (MH) | AMK, GEN |  | CAZ, CIP, CRO, DOX, IPM, MEM, SAM, SXT, TET | YES | *bla*_OXA-23-like_ |
| AB4 | 22 | F | ETA | ICUA (MH) | AMK, GEN | SAM | CAZ, CIP, CRO, DOX, IPM, MEM, SXT, TET | NO | *bla*_OXA-23-like_ |
| AB5 | 53 | M | ETA | ICUA (MH) | GEN |  | AMK, CAZ, CIP, CRO, DOX, IPM, MEM, SAM, SXT, TET | NO | *bla*_OXA-23-like_  *bla*_NDM_ |
| AB6 | 29 | F | ETA | ICUA (MH) | AMK, DOX, SXT |  | CAZ, CIP, CRO, GEN, IPM, MEM, SAM, TET | NO | *bla*_OXA-23-like_ |
| AB7 | 49 | M | ETA | ICUA (MH) | DOX, SXT |  | AMK, CAZ, CIP, CRO, GEN, IPM, MEM, SAM, TET | NO | *bla*_OXA-23-like_ |
| AB8 | 75 | M | ETA | ICUA (MH) |  | SXT | AMK, CAZ, CIP, CRO, DOX, GEN, IPM, MEM, SAM, TET | NO | *bla*_OXA-23-like_  *bla*_NDM_ |
| AB9 | 93 | F | ETA | ICUA (MH) | AMK, DOX, SXT |  | CAZ, CI, CRO, GEN, IPM, MEM, SAM, TET | NO | *bla*_OXA-23-like_ |
| AB10 | 48 | F | ETA | ICUA (MH) | AMK, GEN | SXT | CAZ, CIP, CRO, DOX, IPM, MEM, SAM, TET | NO | *bla*_OXA-23-like_ |
| AB11 | 42 | M | ETA | ICUA (MH) | AMK, GEN |  | CAZ, CIP, CRO, DOX, IPM, MEM, SAM, SXT, TET | NO | *bla*_OXA-23-like_ |
| AB12 | 44 | F | ETA | ICUA (MH) | AMK, GEN, SXT |  | CAZ, CIP, CRO, DOX, IPM, MEM, SAM, TET | UNKNOWN | *bla*_OXA-23-like_ |
| AB13 | 80 | F | ETA | ICUA (MH) | AMK, GEN | SXT | CAZ, CIP, CRO, DOX, IPM, MEM, SAM, TET | NO | *bla*_OXA-23-like_ |
| AB14 | 76 | M | ETA | ICUA (MH) | AMK, GEN |  | CAZ, CIP, CRO, DOX, IPM, MEM, SAM, SXT, TET | NO | *bla*_OXA-23-like_ |
| AB15 | 72 | F | ETA | ICUA (MH) | AMK, GEN | SXT | CAZ, CIP, CRO, DOX, IPM, MEM, SAM, TET | NO | *bla*_OXA-23-like_ |
| AB16 | 55 | F | ETA | ICUA (MH) | AMK, DOX, SX |  | CAZ, CIP, CRO, GEN, IPM, MEM, SAM, TET | NO | *bla*_OXA-23-like_ |
| AB17 | 75 | M | ETA | ICUA (MH) | AMK, GEN, SXT |  | CAZ, CIP, CRO, DOX, IPM, MEM, SAM, TET | NO | *bla*_OXA-23-like_ |
| AB18 | 73 | F | ETA | ICUA (MH) | AMK, GEN | SAM, SXT | CAZ, CIP, CRO, DOX, IPM, MEM, TET | NO | *bla*_OXA-23-like_ |
| AB19 | 38 | M | ETA | ICUA (MH) | AMK, DOX, SXT |  | CAZ, CI, CRO, GEN, IPM, MEM, SAM, TET | NO | *bla*_OXA-23-like_ |
| AB20 | 65 | F | WS | ICUA (MIL) |  | SXT | AMK, CAZ, CIP, CRO, DOX, GEN, IPM, MEM, SAM, TET | UNKNOWN | *bla*_OXA-23-like_ |
| AB21 | 67 | F | WS | GENSUR (MH) | AMK, DOX SXT |  | CAZ, CIP, CRO, GEN, IPM, MEM, SAM, TET | UNKNOWN | *bla*_OXA-23-like_ |
| AB22 | 85 | F | BC | GENMED (MH) | AMK, GEN |  | CAZ, CIP, CRO, DOX, IPM, MEM, SAM, SXT, TET | NO | *bla*_OXA-23-like_ |

*AMK, amikacin; CAZ, ceftazidime; CIP, ciprofloxacin; GEN, gentamicin; CRO, ceftriaxone; DOX, doxycycline; IPM, imipenem; MEM, meropenem; SAM, ampicillin/sulbactam; SXT, trimethoprim/sulphamethoxazole; TET, tetracycline*

*ICUA, Adult ICU; GENSUR, General Surgery; GENMED, General Medicine; MH, Mahosot Hospital, Vientiane; MIL, Military Hospital, Vientiane*

*BC, blood culture; ETA, endotracheal aspirate; WS, wound swab*
